# (3,6-Dimethyl-1,2,4,5-tetra­zine-1,4-di­yl)bis­[(morpholin-4-yl)methanone]

**DOI:** 10.1107/S1600536812004849

**Published:** 2012-02-10

**Authors:** Na-Bo Sun, Yan-Mei Guo, Guo-Wu Rao

**Affiliations:** a College of Biology and Environmental Engineering, Zhejiang Shuren University, Hangzhou 310015, People’s Republic of China; b College of Pharmaceutical Science, Zhejiang University of Technology, Hangzhou 310014, People’s Republic of China

## Abstract

In the title mol­ecule, C_14_H_22_N_6_O_4_, the amide-substituted N atoms of the tetra­zine ring deviate from the approximate plane of the four other atoms in the ring by 0.160 (2) and 0.243 (2) Å, forming a slight boat conformation. The morpholine rings are in chair conformations.

## Related literature

For chemical reactions of 1,2,4,5-tetra­zine derivatives, see: Domingo *et al.* (2009 ▶); Lorincz *et al.* (2010 ▶). For their bio­logical activities, see: Devaraj *et al.* (2009 ▶); Eremeev *et al.* (1978 ▶, 1980 ▶); Han *et al.* (2010 ▶); Neunhoeffer (1984 ▶); Sauer (1996 ▶). For anti-tumor activity of 1,2,4,5-tetra­zine derivatives, see: Hu *et al.* (2002 ▶, 2004 ▶); Rao & Hu, (2005 ▶, 2006 ▶). For details of the synthesis, see: Hu *et al.* (2004 ▶); Skorianetz & Kováts (1970 ▶, 1971 ▶); Sun *et al.* (2003 ▶). For standard bond lengths, see: Allen *et al.* (1987 ▶).
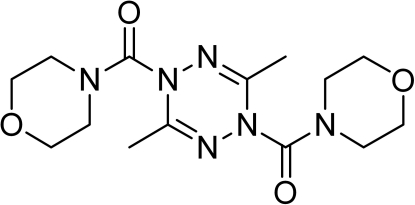


## Experimental

### 

#### Crystal data

C_14_H_22_N_6_O_4_
*M* *_r_* = 338.38
Monoclinic, 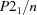

*a* = 15.285 (3) Å
*b* = 6.5977 (14) Å
*c* = 16.729 (4) Å
β = 106.576 (3)°
*V* = 1617.0 (6) Å^3^
*Z* = 4
Mo *K*α radiation
μ = 0.10 mm^−1^
*T* = 298 K
0.55 × 0.42 × 0.28 mm

#### Data collection

Bruker SMART CCD diffractometer
Absorption correction: multi-scan (*SADABS*; Bruker, 1997 ▶) *T*_min_ = 0.944, *T*_max_ = 0.971
9248 measured reflections
3714 independent reflections
2908 reflections with *I* > 2σ(*I*)
*R*_int_ = 0.020

#### Refinement

*R*[*F* ^2^ > 2σ(*F* ^2^)] = 0.049
*wR*(*F* ^2^) = 0.142
*S* = 1.03
3714 reflections
220 parameters
H-atom parameters constrained
Δρ_max_ = 0.27 e Å^−3^
Δρ_min_ = −0.27 e Å^−3^

### 

Data collection: *SMART* (Bruker, 1997 ▶); cell refinement: *SAINT* (Bruker, 1997 ▶); data reduction: *SAINT*; program(s) used to solve structure: *SHELXS97* (Sheldrick, 2008 ▶); program(s) used to refine structure: *SHELXL97* (Sheldrick, 2008 ▶); molecular graphics: *ORTEP-3 for Windows* (Farrugia, 1997 ▶); software used to prepare material for publication: *WinGX* (Farrugia, 1999 ▶).

## Supplementary Material

Crystal structure: contains datablock(s) I, global. DOI: 10.1107/S1600536812004849/lh5412sup1.cif

Supplementary material file. DOI: 10.1107/S1600536812004849/lh5412Isup2.cdx

Structure factors: contains datablock(s) I. DOI: 10.1107/S1600536812004849/lh5412Isup3.hkl

Supplementary material file. DOI: 10.1107/S1600536812004849/lh5412Isup4.cml

Additional supplementary materials:  crystallographic information; 3D view; checkCIF report

## Figures and Tables

**Fig. 1.**
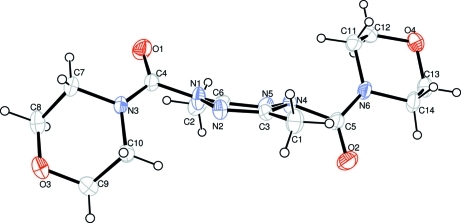
The molecular structure of (I), shown with 30% probability displacement ellipsoids.

**Fig. 2.**
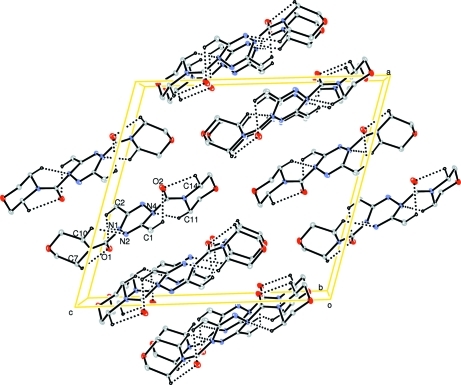
The crystal packing of (I). Hydrogen bonds are shown as dashed lines. H atoms not involved in hydrogen bonding were omitted for clarity.

## supplementary crystallographic information

### Comment

Tetrazine derivatives have high activity in chemical reactions (Domingo *et al.*, 2009; Lorincz *et al.*, 2010), and have been widely used in pesticides and medicines (Devaraj *et al.*, 2009; Eremeev *et al.*, 1978, 1980; Han *et al.*, 2010; Neunhoeffer, 1984; Sauer, 1996). In a continuation of our studies of antitumor activities in 1,2,4,5-tetrazine derivatives (Hu *et al.*, 2002, 2004; Rao & Hu, 2005, 2006), we have obtained a yellow crystalline compound, (I). The structure was confirmed by single-crystal X-ray diffraction.

The molecular structure of (I) is illustrated in Fig. 1. The N2═C3 [1.2730 (17) Å] and N5═C6 [1.2748 (18) Å] bonds lengths are typical for double bonds, as are the C3—N4 [1.3821 (17) Å], N4—N5 [1.4235 (17) Å], C6—N1 [1.3728 (17) Å] and N1—N2 [1.4207 (16) Å] bond lengths (Allen *et al.*, 1987). The tetrazine ring is a 1,4-dihydro structure with the N-substituted groups at the 1,4-positions.

In (I), atoms N2, C3, N5 and C6 are approximately planar, with the largest deviation from this plane being 0.0137 (6) Å. Atoms N1 and N4 deviate from this plane by 0.1604 (21) and 0.2429 (20) Å, respectively. The dihedral angle between the N2/C3/N5/C6 plane and the N1/N2/C6 plane is 13.37 (24)°, and between the N2/C3/N5/C6 plane and the N4/N5/C3 plane is 19.79 (21)°. The tetrazine ring has a slight boat conformation. Atoms C7, C8, C9 and C10 are approximately planar, with the largest deviation from this plane being 0.0114 (9) Å. Atoms O3 and N3 deviate from this plane by 0.6616 (23) and -0.5938 (21) Å, respectively. Atoms C11, C12, C13 and C14 are approximately planar, with the largest deviation from this plane being 0.0137 (9) Å. Atoms O4 and N6 deviate from this plane by 0.6576 (20) and -0.5953 (21) Å, respectively. The two morpholine rings exhibit chair conformations.

### Experimental

The title compound was the product of the reaction of bis(trichloromethyl) carbonate, morpholine, and 3,6-dimethyl-1,6-dihydro-1,2,4,5-tetrazine according to the procedure (Hu *et al.*, 2004; Sun *et al.*, 2003; Skorianetz & Kováts, 1970, 1971). A solution of the title compound in acetone was concentrated gradually at room temperature to afford yellow blocks.

### Refinement

H atoms were included in calculated positions and refined using a riding model. H atoms were given isotropic displacement parameters equal to 1.2 (or 1.5 for methyl H atoms) times the equivalent isotropic displacement parameters of their parent atoms, and C—H distances were set to 0.96Å for methyl H atoms and 0.97 Å for methylene H atoms.

### Crystal data

**Table:**

C_14_H_22_N_6_O_4_	*F*(000) = 720
*M**_r_* = 338.38	*D*_x_ = 1.390 Mg m^−^^3^
Monoclinic, *P*2_1_/*n*	Mo *K*α radiation, λ = 0.71073 Å
Hall symbol: -P 2yn	Cell parameters from 6920 reflections
*a* = 15.285 (3) Å	θ = 2.9–28.2°
*b* = 6.5977 (14) Å	µ = 0.10 mm^−^^1^
*c* = 16.729 (4) Å	*T* = 298 K
β = 106.576 (3)°	Block, yellow
*V* = 1617.0 (6) Å^3^	0.55 × 0.42 × 0.28 mm
*Z* = 4	

### Data collection

**Table:**

Bruker SMART CCD diffractometer	3714 independent reflections
Radiation source: fine-focus sealed tube	2908 reflections with *I* > 2σ(*I*)
Graphite monochromator	*R*_int_ = 0.020
φ and ω scans	θ_max_ = 28.3°, θ_min_ = 2.1°
Absorption correction: multi-scan (*SADABS*; Bruker, 1997)	*h* = −15→20
*T*_min_ = 0.944, *T*_max_ = 0.971	*k* = −8→8
9248 measured reflections	*l* = −18→21

### Refinement

**Table:**

Refinement on *F*^2^	Secondary atom site location: difference Fourier map
Least-squares matrix: full	Hydrogen site location: inferred from neighbouring sites
*R*[*F*^2^ > 2σ(*F*^2^)] = 0.049	H-atom parameters constrained
*wR*(*F*^2^) = 0.142	*w* = 1/[σ^2^(*F*_o_^2^) + (0.0823*P*)^2^ + 0.2523*P*] where *P* = (*F*_o_^2^ + 2*F*_c_^2^)/3
*S* = 1.03	(Δ/σ)_max_ < 0.001
3714 reflections	Δρ_max_ = 0.27 e Å^−^^3^
220 parameters	Δρ_min_ = −0.27 e Å^−^^3^
0 restraints	Extinction correction: *SHELXL97* (Sheldrick, 2008), Fc^*^=kFc[1+0.001xFc^2^λ^3^/sin(2θ)]^-1/4^
Primary atom site location: structure-invariant direct methods	Extinction coefficient: 0.036 (3)

### Special details

**Table:**

Geometry. All esds (except the esd in the dihedral angle between two l.s. planes) are estimated using the full covariance matrix. The cell esds are taken into account individually in the estimation of esds in distances, angles and torsion angles; correlations between esds in cell parameters are only used when they are defined by crystal symmetry. An approximate (isotropic) treatment of cell esds is used for estimating esds involving l.s. planes.
Refinement. Refinement of F^2^ against ALL reflections. The weighted R-factor wR and goodness of fit S are based on F^2^, conventional R-factors R are based on F, with F set to zero for negative F^2^. The threshold expression of F^2^ > 2sigma(F^2^) is used only for calculating R-factors(gt) etc. and is not relevant to the choice of reflections for refinement. R-factors based on F^2^ are statistically about twice as large as those based on F, and R- factors based on ALL data will be even larger.

### Fractional atomic coordinates and isotropic or equivalent isotropic displacement parameters (Å^2^)

**Table:**

	*x*	*y*	*z*	*U*_iso_*/*U*_eq_	
N2	0.68618 (8)	0.70860 (18)	0.11259 (7)	0.0374 (3)	
N4	0.59388 (8)	0.60296 (19)	0.19430 (7)	0.0409 (3)	
N5	0.55633 (9)	0.43942 (18)	0.13981 (8)	0.0450 (3)	
N1	0.66936 (9)	0.51243 (19)	0.07644 (8)	0.0456 (3)	
O4	0.49306 (9)	0.2853 (2)	0.41742 (7)	0.0586 (3)	
O3	0.74085 (9)	0.7091 (2)	−0.18200 (7)	0.0620 (4)	
N6	0.53166 (9)	0.5633 (2)	0.30490 (8)	0.0444 (3)	
C6	0.59824 (9)	0.3973 (2)	0.08605 (8)	0.0359 (3)	
N3	0.75197 (8)	0.57709 (19)	−0.01932 (7)	0.0416 (3)	
O1	0.75909 (9)	0.27148 (18)	0.04345 (9)	0.0643 (4)	
C3	0.64652 (9)	0.7449 (2)	0.16842 (8)	0.0333 (3)	
O2	0.48046 (10)	0.8161 (2)	0.21322 (9)	0.0764 (5)	
C4	0.72971 (10)	0.4435 (2)	0.03193 (8)	0.0397 (3)	
C5	0.52962 (10)	0.6716 (2)	0.23773 (9)	0.0415 (3)	
C11	0.59485 (10)	0.3983 (2)	0.33971 (10)	0.0461 (4)	
H11A	0.6405	0.4442	0.3895	0.055*	
H11B	0.6258	0.3549	0.2994	0.055*	
C12	0.54193 (11)	0.2249 (3)	0.36082 (10)	0.0492 (4)	
H12A	0.4994	0.1738	0.3101	0.059*	
H12B	0.5836	0.1161	0.3854	0.059*	
C7	0.82145 (11)	0.5206 (3)	−0.06011 (10)	0.0490 (4)	
H7A	0.8456	0.3874	−0.0413	0.059*	
H7B	0.8714	0.6170	−0.0455	0.059*	
C10	0.70416 (11)	0.7647 (2)	−0.05175 (9)	0.0461 (4)	
H10A	0.7453	0.8788	−0.0350	0.055*	
H10B	0.6533	0.7839	−0.0287	0.055*	
C14	0.47410 (12)	0.6227 (3)	0.35760 (11)	0.0531 (4)	
H14A	0.4279	0.7179	0.3278	0.064*	
H14B	0.5111	0.6889	0.4076	0.064*	
C1	0.66582 (12)	0.9416 (3)	0.21394 (11)	0.0535 (4)	
H1A	0.6875	0.9164	0.2728	0.080*	
H1B	0.6109	1.0208	0.2020	0.080*	
H1C	0.7115	1.0143	0.1963	0.080*	
C13	0.42906 (12)	0.4385 (3)	0.38095 (11)	0.0548 (4)	
H13A	0.3958	0.4774	0.4200	0.066*	
H13B	0.3854	0.3847	0.3315	0.066*	
C2	0.56523 (12)	0.2236 (3)	0.02845 (12)	0.0594 (5)	
H2A	0.5571	0.2667	−0.0280	0.089*	
H2B	0.5081	0.1761	0.0345	0.089*	
H2C	0.6093	0.1159	0.0418	0.089*	
C9	0.66960 (12)	0.7561 (3)	−0.14556 (10)	0.0588 (5)	
H9A	0.6222	0.6540	−0.1618	0.071*	
H9B	0.6429	0.8858	−0.1666	0.071*	
C8	0.77950 (13)	0.5188 (3)	−0.15316 (10)	0.0581 (5)	
H8A	0.8259	0.4854	−0.1803	0.070*	
H8B	0.7326	0.4153	−0.1678	0.070*	

### Atomic displacement parameters (Å^2^)

**Table:**

	*U*^11^	*U*^22^	*U*^33^	*U*^12^	*U*^13^	*U*^23^
N2	0.0443 (6)	0.0343 (6)	0.0378 (6)	−0.0095 (5)	0.0183 (5)	−0.0074 (5)
N4	0.0480 (7)	0.0397 (7)	0.0428 (6)	−0.0052 (5)	0.0255 (5)	−0.0037 (5)
N5	0.0469 (7)	0.0330 (6)	0.0625 (8)	−0.0085 (5)	0.0278 (6)	−0.0075 (5)
N1	0.0601 (8)	0.0379 (7)	0.0506 (7)	−0.0152 (6)	0.0346 (6)	−0.0145 (5)
O4	0.0659 (7)	0.0694 (8)	0.0489 (6)	0.0032 (6)	0.0302 (6)	0.0145 (6)
O3	0.0741 (8)	0.0787 (9)	0.0390 (6)	0.0023 (7)	0.0255 (6)	0.0062 (6)
N6	0.0515 (7)	0.0449 (7)	0.0471 (7)	0.0098 (6)	0.0306 (6)	0.0054 (5)
C6	0.0375 (7)	0.0288 (7)	0.0417 (7)	−0.0002 (5)	0.0119 (6)	−0.0014 (5)
N3	0.0471 (7)	0.0461 (7)	0.0390 (6)	0.0091 (5)	0.0239 (5)	0.0036 (5)
O1	0.0768 (8)	0.0459 (7)	0.0819 (9)	0.0174 (6)	0.0417 (7)	0.0147 (6)
C3	0.0353 (6)	0.0348 (7)	0.0297 (6)	−0.0013 (5)	0.0089 (5)	−0.0013 (5)
O2	0.0777 (9)	0.0787 (9)	0.0906 (10)	0.0378 (8)	0.0525 (8)	0.0386 (8)
C4	0.0438 (7)	0.0405 (8)	0.0376 (7)	0.0007 (6)	0.0163 (6)	−0.0037 (6)
C5	0.0431 (7)	0.0416 (8)	0.0456 (8)	0.0033 (6)	0.0221 (6)	0.0017 (6)
C11	0.0434 (8)	0.0514 (9)	0.0468 (8)	0.0064 (7)	0.0182 (6)	0.0062 (7)
C12	0.0541 (9)	0.0496 (9)	0.0457 (8)	0.0062 (7)	0.0173 (7)	0.0079 (7)
C7	0.0487 (8)	0.0588 (10)	0.0483 (8)	0.0086 (7)	0.0278 (7)	0.0008 (7)
C10	0.0564 (9)	0.0445 (8)	0.0424 (8)	0.0095 (7)	0.0223 (7)	0.0053 (6)
C14	0.0653 (10)	0.0531 (10)	0.0544 (9)	0.0064 (8)	0.0386 (8)	−0.0022 (7)
C1	0.0623 (10)	0.0481 (9)	0.0552 (9)	−0.0113 (8)	0.0248 (8)	−0.0196 (7)
C13	0.0541 (9)	0.0674 (11)	0.0531 (9)	0.0012 (8)	0.0316 (8)	0.0028 (8)
C2	0.0529 (9)	0.0473 (9)	0.0776 (12)	−0.0106 (7)	0.0179 (9)	−0.0261 (8)
C9	0.0598 (10)	0.0707 (12)	0.0452 (9)	0.0083 (9)	0.0138 (8)	0.0096 (8)
C8	0.0704 (11)	0.0663 (11)	0.0470 (9)	−0.0035 (9)	0.0318 (8)	−0.0119 (8)

### Geometric parameters (Å, º)

**Table:**

N2—C3	1.2730 (17)	C11—H11B	0.9700
N2—N1	1.4207 (16)	C12—H12A	0.9700
N4—C3	1.3821 (17)	C12—H12B	0.9700
N4—N5	1.4235 (17)	C7—C8	1.505 (2)
N4—C5	1.4509 (17)	C7—H7A	0.9700
N5—C6	1.2748 (18)	C7—H7B	0.9700
N1—C6	1.3728 (17)	C10—C9	1.507 (2)
N1—C4	1.4155 (17)	C10—H10A	0.9700
O4—C13	1.418 (2)	C10—H10B	0.9700
O4—C12	1.4201 (19)	C14—C13	1.502 (2)
O3—C8	1.413 (2)	C14—H14A	0.9700
O3—C9	1.426 (2)	C14—H14B	0.9700
N6—C5	1.3245 (19)	C1—H1A	0.9600
N6—C11	1.4620 (19)	C1—H1B	0.9600
N6—C14	1.4653 (17)	C1—H1C	0.9600
C6—C2	1.490 (2)	C13—H13A	0.9700
N3—C4	1.3392 (18)	C13—H13B	0.9700
N3—C10	1.4609 (19)	C2—H2A	0.9600
N3—C7	1.4650 (17)	C2—H2B	0.9600
O1—C4	1.2158 (18)	C2—H2C	0.9600
C3—C1	1.4911 (19)	C9—H9A	0.9700
O2—C5	1.2104 (19)	C9—H9B	0.9700
C11—C12	1.500 (2)	C8—H8A	0.9700
C11—H11A	0.9700	C8—H8B	0.9700
			
C3—N2—N1	114.67 (11)	C8—C7—H7B	109.8
C3—N4—N5	118.51 (11)	H7A—C7—H7B	108.2
C3—N4—C5	118.88 (12)	N3—C10—C9	110.14 (13)
N5—N4—C5	110.52 (11)	N3—C10—H10A	109.6
C6—N5—N4	115.15 (11)	C9—C10—H10A	109.6
C6—N1—C4	122.73 (12)	N3—C10—H10B	109.6
C6—N1—N2	120.49 (11)	C9—C10—H10B	109.6
C4—N1—N2	116.78 (11)	H10A—C10—H10B	108.1
C13—O4—C12	110.04 (12)	N6—C14—C13	109.79 (13)
C8—O3—C9	110.07 (13)	N6—C14—H14A	109.7
C5—N6—C11	126.36 (12)	C13—C14—H14A	109.7
C5—N6—C14	119.56 (13)	N6—C14—H14B	109.7
C11—N6—C14	113.66 (12)	C13—C14—H14B	109.7
N5—C6—N1	122.43 (12)	H14A—C14—H14B	108.2
N5—C6—C2	118.58 (13)	C3—C1—H1A	109.5
N1—C6—C2	118.90 (13)	C3—C1—H1B	109.5
C4—N3—C10	127.14 (12)	H1A—C1—H1B	109.5
C4—N3—C7	118.76 (13)	C3—C1—H1C	109.5
C10—N3—C7	113.26 (12)	H1A—C1—H1C	109.5
N2—C3—N4	122.96 (12)	H1B—C1—H1C	109.5
N2—C3—C1	118.17 (12)	O4—C13—C14	112.17 (14)
N4—C3—C1	118.54 (12)	O4—C13—H13A	109.2
O1—C4—N3	124.47 (13)	C14—C13—H13A	109.2
O1—C4—N1	118.89 (13)	O4—C13—H13B	109.2
N3—C4—N1	116.62 (13)	C14—C13—H13B	109.2
O2—C5—N6	125.04 (13)	H13A—C13—H13B	107.9
O2—C5—N4	121.32 (13)	C6—C2—H2A	109.5
N6—C5—N4	113.63 (12)	C6—C2—H2B	109.5
N6—C11—C12	108.81 (12)	H2A—C2—H2B	109.5
N6—C11—H11A	109.9	C6—C2—H2C	109.5
C12—C11—H11A	109.9	H2A—C2—H2C	109.5
N6—C11—H11B	109.9	H2B—C2—H2C	109.5
C12—C11—H11B	109.9	O3—C9—C10	111.70 (14)
H11A—C11—H11B	108.3	O3—C9—H9A	109.3
O4—C12—C11	111.39 (14)	C10—C9—H9A	109.3
O4—C12—H12A	109.4	O3—C9—H9B	109.3
C11—C12—H12A	109.4	C10—C9—H9B	109.3
O4—C12—H12B	109.4	H9A—C9—H9B	107.9
C11—C12—H12B	109.4	O3—C8—C7	111.07 (14)
H12A—C12—H12B	108.0	O3—C8—H8A	109.4
N3—C7—C8	109.40 (13)	C7—C8—H8A	109.4
N3—C7—H7A	109.8	O3—C8—H8B	109.4
C8—C7—H7A	109.8	C7—C8—H8B	109.4
N3—C7—H7B	109.8	H8A—C8—H8B	108.0
			
C3—N4—N5—C6	23.26 (19)	C11—N6—C5—O2	−174.99 (17)
C5—N4—N5—C6	165.46 (12)	C14—N6—C5—O2	−2.9 (3)
C3—N2—N1—C6	16.02 (19)	C11—N6—C5—N4	4.4 (2)
C3—N2—N1—C4	−163.17 (13)	C14—N6—C5—N4	176.51 (13)
N4—N5—C6—N1	−5.6 (2)	C3—N4—C5—O2	44.9 (2)
N4—N5—C6—C2	177.96 (14)	N5—N4—C5—O2	−97.15 (18)
C4—N1—C6—N5	164.79 (14)	C3—N4—C5—N6	−134.55 (14)
N2—N1—C6—N5	−14.4 (2)	N5—N4—C5—N6	83.42 (15)
C4—N1—C6—C2	−18.8 (2)	C5—N6—C11—C12	−134.69 (16)
N2—N1—C6—C2	162.09 (14)	C14—N6—C11—C12	52.82 (18)
N1—N2—C3—N4	2.27 (19)	C13—O4—C12—C11	61.50 (17)
N1—N2—C3—C1	175.66 (13)	N6—C11—C12—O4	−57.44 (17)
N5—N4—C3—N2	−22.2 (2)	C4—N3—C7—C8	117.84 (16)
C5—N4—C3—N2	−161.22 (13)	C10—N3—C7—C8	−52.37 (18)
N5—N4—C3—C1	164.45 (13)	C4—N3—C10—C9	−118.42 (16)
C5—N4—C3—C1	25.42 (19)	C7—N3—C10—C9	50.80 (18)
C10—N3—C4—O1	163.86 (16)	C5—N6—C14—C13	135.96 (16)
C7—N3—C4—O1	−4.8 (2)	C11—N6—C14—C13	−50.99 (19)
C10—N3—C4—N1	−17.6 (2)	C12—O4—C13—C14	−59.39 (18)
C7—N3—C4—N1	173.67 (13)	N6—C14—C13—O4	53.49 (19)
C6—N1—C4—O1	−44.3 (2)	C8—O3—C9—C10	59.94 (19)
N2—N1—C4—O1	134.89 (16)	N3—C10—C9—O3	−53.83 (19)
C6—N1—C4—N3	137.12 (15)	C9—O3—C8—C7	−61.71 (18)
N2—N1—C4—N3	−43.71 (18)	N3—C7—C8—O3	57.37 (19)

### Hydrogen-bond geometry (Å, º)

**Table:**

*D*—H···*A*	*D*—H	H···*A*	*D*···*A*	*D*—H···*A*
C14—H14*A*···O2	0.97	2.37	2.758 (2)	103
C1—H1*B*···O2	0.96	2.46	2.949 (2)	111
C2—H2*C*···O1	0.96	2.50	2.919 (2)	106
C7—H7*A*···O1	0.97	2.33	2.748 (2)	105
C10—H10*B*···N1	0.97	2.47	2.881 (2)	105
C10—H10*B*···N2	0.97	2.33	2.8640 (19)	114
C11—H11*B*···N4	0.97	2.35	2.7787 (19)	106

## References

[bb1] Allen, F. H., Kennard, O., Watson, D. G., Brammer, L. & Orpen, A. G. (1987). *J. Chem. Soc. Perkin Trans. 2*, pp. S1–19.

[bb2] Bruker (1997). *SMART*, *SAINT* and *SADABS* Bruker AXS Inc., Madison, Wisconsin, USA.

[bb3] Devaraj, N. K., Upadhyay, R., Haun, J. B., Hilderbrand, S. A. & Weissleder, R. (2009). *Angew. Chem. Int. Ed.* **48**, 7013–7016. 10.1002/anie.200903233 PMC2790075 19697389

[bb4] Domingo, L. R., Picher, M. T. & Saez, J. A. (2009). *J. Org. Chem.* **74**, 2726–2735. 10.1021/jo802822u 19260699

[bb5] Eremeev, A. V., Tikhomirova, D. A. & Zidermane, A. (1980). USSR Patent No. 686336.

[bb6] Eremeev, A. V., Tikhomirv, D. A., Tyusheva, V. A. & Liepins, F. (1978). *Khim. Geterotsikl. Soedin.* **6**, 753–757.

[bb7] Farrugia, L. J. (1997). *J. Appl. Cryst.* **30**, 565.

[bb8] Farrugia, L. J. (1999). *J. Appl. Cryst.* **32**, 837–838.

[bb9] Han, H. S., Devaraj, N. K., Lee, J., Hilderbrand, S. A., Weissleder, R. & Bawendi, M. G. (2010). *J. Am. Chem. Soc.* **132**, 7838–7839. 10.1021/ja101677r PMC2892974 20481508

[bb10] Hu, W. X., Rao, G. W. & Sun, Y. Q. (2004). *Bioorg. Med. Chem. Lett.* **14**, 1177–1181. 10.1016/j.bmcl.2003.12.056 14980660

[bb11] Hu, W. X., Sun, Y. Q., Yuan, Q. & Yang, Z. Y. (2002). *Chem. J. Chin. Univ.* **23**, 1877–1881.

[bb12] Lorincz, K., Kotschy, A., Tammiku-Taul, J., Sikk, L. & Burk, P. (2010). *J. Org. Chem.* **75**, 6196–6200. 10.1021/jo101119n 20735149

[bb13] Neunhoeffer, H. (1984). *Comprehensive Heterocyclic Chemistry*, Vol. 3, edited by A. R. Katritzky, 1st ed., pp. 531–572. Frankfurt: Pergamon.

[bb14] Rao, G. W. & Hu, W. X. (2005). *Bioorg. Med. Chem. Lett.* **15**, 3174–3176. 10.1016/j.bmcl.2005.03.122 15908202

[bb15] Rao, G. W. & Hu, W. X. (2006). *Bioorg. Med. Chem. Lett.* **16**, 3702–3705. 10.1016/j.bmcl.2006.04.066 16709456

[bb16] Sauer, J. (1996). *Comprehensive Heterocyclic Chemistry*, Vol. 6, edited by A. J. Boulton, 2nd ed., pp. 901–955. Oxford: Elsevier.

[bb17] Sheldrick, G. M. (2008). *Acta Cryst.* A**64**, 112–122. 10.1107/S0108767307043930 18156677

[bb18] Skorianetz, W. & Kováts, E. Sz. (1970). *Helv. Chim. Acta*, **53**, 251–262.

[bb19] Skorianetz, W. & Kováts, E. Sz. (1971). *Helv. Chim. Acta*, **54**, 1922–1939.

[bb20] Sun, Y. Q., Hu, W. X. & Yuan, Q. (2003). *Synth. Commun.* **33**, 2769–2775.

